# Impact of Genetic Polymorphisms and Biomarkers on the Effectiveness and Toxicity of Treatment of Chronic Myeloid Leukemia and Acute Myeloid Leukemia

**DOI:** 10.3390/jpm12101607

**Published:** 2022-09-29

**Authors:** Carolina Alarcón-Payer, María Del Mar Sánchez Suárez, Alicia Martín Roldán, José Manuel Puerta Puerta, Alberto Jiménez Morales

**Affiliations:** 1 Servicio de Farmacia, Hospital Universitario Virgen de las Nieves, 18014 Granada, Spain; 2 Unidad de Gestión Clínica Hematología y Hemoterapia, Hospital Universitario Virgen de las Nieves, 18014 Granada, Spain

**Keywords:** acute myeloid leukemia, chronic myeloid leukemia, pharmacogenetics, polymorphisms, response, biomarkers, personalized medicine, tyrosine kinase inhibitors

## Abstract

Most malignant hematological diseases are generally a consequence of acquired mutations or rearrangements in cell replication processes. Acute myeloid leukemia (AML) is a clinically and molecularly heterogeneous disease that results from acquired genetic and epigenetic alterations in hematopoietic progenitor cells. Despite the advances made in understanding the pathogenesis of this disease, the overall survival of patients remains very low due to the high relapse rate. Pharmacogenetics and massive sequencing studies have allowed the identification of new recurrent mutations with significant prognostic impact in AML; furthermore, it seems likely that whole genome sequencing will soon become a standard diagnostic test, which will allow the molecular diagnosis of patients. Therefore, it is necessary to develop molecular targets that open new therapeutic perspectives and allow individualized treatment of patients with this aggressive disease. Chronic myeloid leukemia (CML) is the first neoplastic disease for which a characteristic genetic alteration was described. It has, by definition, a genetic marker, the BCR::ABL1 rearrangement, as a consequence of the t9;22(q34;q11) translocation. Its study is essential for the diagnosis of this entity and also for monitoring the response to treatment. Drugs known as tyrosine kinase inhibitors (TKIs) that target the BCR::ABL1 protein (oral targeted therapy) are the conventional treatment of CML, representing a change of paradigm in the management of oncohematological patients.

## 1. Introduction

Chronic myeloid leukemia (CML), also known as myelogenous or chronic granulocytic leukemia, is defined as a clonal hematologic malignant neoplastic disease of pluripotent stem cells classified according to the latest WHO classification of 2008 within the group of chronic myeloproliferative neoplasms (CMPN) [1]. The presence of a reciprocal translocation between chromosomes 9 and 22 in the hematopoietic cells of more than 90% of diagnosed patients is characteristic of CML, leading to the formation of a clearly shortened long arm of one of the chromosomes of pair 22, known as the Philadelphia chromosome (Ph). The molecular impact of the exchange of genetic material between chromosomes 9 and 22 is the *BCR::ABL1* oncogene, which results in the synthesis of a protein with increased tyrosine kinase activity, the BCR::ABL1 protein, whose resulting leukemic activity leads to a significant expansion of the erythroid, granulocytic and megakaryocytic progenitor populations, with reduced sensitivity of the progenitors to the regulation of the process of hematopoiesis [2]. This is the first neoplasm in which the responsible genetic alteration has been identified; it is unusual in cancer for a single oncogene to produce a neoplastic process [3]. The cause of the genetic translocation is unknown; in some cases, it has been attributed to environmental factors, such as ionizing radiation or chemical compounds such as benzene derivatives, but the origin is not really known. The excessive proliferation of the myeloid cell line in the bone marrow (BM) is reflected in an increase in peripheral blood leukocytes (PB) at different stages of maturation and with apparent morphological normality. CML is a rare disease affecting all races, with a slight predominance in males. It accounts for 15–20% of all leukemias. It can occur at any age and is rare in children. The highest number of cases appears between 40 and 60 years of age. It is a disease of low incidence (1–1.5 cases per 100,000 inhabitants/year), but currently, after treatment with TKI, the prevalence of the disease doubles every five years [4,5].

Diagnosis of CML requires the presence of the reciprocal translocation between chromosomes 9 and 22, t(9;22)(q34;q11.2), which results in the chimeric *BCR::ABL1* fusion gene. A thorough initial evaluation of both the disease and the patient is necessary. Regarding the characteristics of the disease, a good morphological and genetic study is essential. It is required to pay special attention to comorbidities that can be aggravated by the treatments. We have helpful tools to predict the prognosis and make therapeutic decisions. Therefore, it is advisable to use a prognostic score; the most widely used in our setting is the SOKAL index. In 40% of the cases, the disease is diagnosed by chance when performing a hemogram for other reasons and in asymptomatic subjects. In 50% of patients, mild to moderate symptoms appear, such as asthenia, anorexia, weight loss, night sweats, spleen enlargement, and anemia. About 85% are diagnosed in the chronic phase of the disease. It is characterized clinically by a bi- or triphasic course. The patient may present in any of these phases, although the most common is the debut as chronic phase (CP), in which the manifestations derived from myeloproliferation (constitutional symptoms, leukocytosis, splenomegaly) are easily controlled with different therapeutic agents, allowing patients to lead a practically normal life. However, the natural evolution of the disease leads to the appearance, after a variable period of between 3 and 6 years, of the terminal phase or blast crisis (BC), characterized by a clinical picture of bone marrow failure, similar to that of acute leukemias and generally refractory to treatment [6]. In about half of the cases, the transition of CML from CP to BC is not abrupt, with an intermediate period, the acceleration phase (AF), whose duration rarely exceeds one year, being interspersed between the two phases. In this phase, patients present a progressive deterioration of their general condition, with the appearance of constitutional symptoms, persistent bone pain, and progressive growth of the spleen despite treatment. The evolution of clinical and analytical parameters defines the transition from one phase to another [7].

Acute myeloid leukemia (AML) is a clonal heterogeneous disease resulting from the clonal expansion of immature cells of myeloid lineage (blasts), which infiltrate the BM, peripheral blood, and other tissues. Most cases of AML are of unknown etiology. However, a higher incidence has been reported in patients with certain chromosomal alterations (Fanconi anemia, Down syndrome, etc.) or with a history of myelodysplastic syndrome or chronic myeloproliferative syndrome. It has also been related to some environmental factors such as ionizing radiation, exposure to benzenes, and previous treatment with alkylating agents or topoisomerase inhibitors [8]. AML is the most frequent acute leukemia in adults, accounting for 40% of all leukemias in the western world. Its incidence is estimated at 3.5 new cases per 100,000 inhabitants per year and increases with age, reaching rates of 12.6 cases per 100,000 inhabitants per year in patients over 65 years of age. The median age at diagnosis is 64 years, and most patients are in the 60–75 age range [9]. The diagnosis of AML requires a detailed study of peripheral blood and BM, with samples obtained by aspiration or biopsy. Classically, the analysis of these samples consisted of a morphological evaluation and the application of histochemical techniques, but this approach is insufficient. Currently, according to the 2016 classification of the World Health Organization, in which the different acute leukemias are classified, both morphological and immunophenotypic, cytogenetic, and molecular analyses are required. For the differential diagnosis, the presence of ≥20% blasts is fundamental, except for myeloid leukemias presenting recurrent cytogenetic alterations such as t(8;21), inv(16), t(16;16), or t(15;17), which are considered acute myeloid leukemias regardless of the percentage of blasts [8]. The clinical symptoms of AML are a reflection of bone marrow failure due to the invasion of leukemic cells, although extramedullary involvement is also possible. The most frequent manifestations at diagnosis are asthenia, weight loss, and pancytopenia [10], leading to increased susceptibility to infection and hemorrhagic complications. Headaches usually characterize the manifestations of extramedullary infiltration due to CNS leukostasis, organomegaly or lymphadenopathy in the liver and spleen, granulocytic sarcoma at the cutaneous level, interstitial infiltrates at the pulmonary level, and gingival hypertrophy or ulcers at the oral level. It also presents with tumor lysis syndrome, leading to metabolic disorders such as hyperuricemia, hypocalcemia, and acute renal failure, triggering arrhythmias and neuromuscular symptoms.

We chose to review these diseases because CML was the first oncohematological disease in history with a specific therapeutic target, the fact that revolutionized targeted oncohematological treatments [11]. In this context, AML, like CML, is undergoing a paradigm shift in the management of oncohematological patients according to the increased use of targeted therapies, which radically improved the prognosis of AML patients [12]. The two pathologies are incomparable except for the myeloid germ lineage. Nevertheless, they are related to each other, since the natural evolution of CML without specific targeted therapy is an inevitable progression to AML, mainly myeloid, and, in some cases, lymphoid [13]. For this reason, we strongly believe that the knowledge of a personalized therapy strategy based on pharmacogenetics can enhance therapeutic outcomes regarding the management of CML and AML. Hence, novel optimized treatments seek to improve efficacy and reduce or at least control toxicity depending on the polymorphisms of each patient [14].

## 2. Materials and Methods

PubMed search included the keywords: “Acute myeloid leukemia” and “Chronic myeloid leukemia” with “polymorphisms”, “toxicity”, and “response”. Data regarding gene, SNP, drug, pathology, population, level of evidence PharmGKB, clinical application, allele, phenotype category (response or toxicity), year of publication, and the number of patients were recorded.

## 3. Treatment of Chronic Myeloid Leukemia (CML) and Acute Myeloid Leukemia (AML)

### 3.1. Chronic Myeloid Leukemia (CML)

The main goal of treatment is to try to cure the disease or prolong the chronic phase during which patients can lead a normal life as much as possible. In the early 1980s, the mainstay of treatment was using drugs such as busulfan, hydroxyurea, interferon, or low-dose cytarabine. Before the introduction of imatinib, life expectancy of 10 years was less than 10%. Thanks to therapeutic advances and the introduction of tyrosine kinase inhibitors (TKIs), CML has been transformed from a fatal disease to a chronic disease. Control of the pathology is achieved in about 95% of patients, which has significant health and pharmacoeconomic impact.

Table 1 lists the differences between tyrosine kinase inhibitors in terms of different targets and therapeutic indications. BCR::ABL1 TKIs block the binding of adenosine triphosphate to BCR::ABL1 tyrosine kinase. Consequently, they inhibit signaling pathways leading to inhibition of cell proliferation and subsequently apoptosis in pathologies with BCR::ABL1 rearrangement without affecting the normal cell. TKIs are the treatment of choice in chronic myeloid leukemia (CML) since the approval of imatinib mesylate (IM) in 2003. Other TKIs are currently available: dasatinib (DA), nilotinib (NI), bosutinib (BO), ponatinib (PO), and asciminib (AS). Asciminib is an allosteric inhibitor that binds a myristoyl site of the BCR::ABL1 protein, locking BCR::ABL1 into an inactive conformation through a mechanism distinct from those for all other ABL kinase inhibitors. Asciminib targets both native and mutated BCR::ABL1, including the gatekeeper T315I mutant [15].

### 3.2. Acute Myeloid Leukemia (AML)

The treatment of AML has evolved considerably over the last decades, and current regimens consider a more individualized treatment. The goal is to achieve complete response (CR) and eliminate minimal residual disease (MRD) to prevent disease recurrence. CR is morphologically defined as the presence of <5% blasts in blood and BM, with the recovery of cell counts in the haemogram. However, remission does not imply a cure, as much tumor mass may remain even after CR is achieved. Except for acute promyelocytic leukemia (APL), which has drugs with an established therapeutic target. Treatment is based on intensive chemotherapy regimens, which do not apply to elderly patients or those with significant comorbidities. The standard regimen is divided into two main phases: an induction phase (to achieve CR) and a post-remission phase (aimed at eradicating the residual leukemic clone) (Table 2). The induction treatment consists of the so-called 3 + 7, which means administering the combination of anthracyclines for 3 days and cytarabine (ARA-C) for 7 days. After one or two cycles of this combination, 60–80% of patients enter CR. Post-remission treatment consists of a first consolidation stage with one cycle equal to induction, followed by two to three cycles of intensification, including intermediate- or high-dose cytarabine. As a final treatment after intensification, autologous or allogeneic hematopoietic stem cell transplantation is the indicated treatment in the first CR, except in the subgroup with a favorable prognosis. Patients who are not candidates for intensive therapies should be approached on an individual basis. Treatment alternatives are based on the administration of low-dose ARA-C, the use of hypomethylating agents such as 5-azacitidine or decitabine, and the inclusion of the patient in clinical trials with new cytotoxic agents or supportive therapy with chemotherapy and general care. These treatment regimens result in less than 50% cure rates for populations eligible for intensive chemotherapy and less than 10% for older patients not eligible for these treatments [21,22]. In addition, unsatisfactory response rates and survival have been reported for conventional chemotherapy in patients with adverse cytogenetic risk or high-risk molecular mutations, such as TP53 [23,24,25]. Advances over the last decade in the genomic and epigenomic spectrum of AML have established new therapeutic targets and the potential development of drugs with more specific mechanisms of action (Table 3).

## 4. General Pharmacogenetics of Chronic Myeloid Leukemia (CML)

### 4.1. Human Genetic Variability and Its Influence on Chronic Myeloid Leukemia (CML)

Inter-individual genetic variability is one of the factors that could influence susceptibility to develop CML and response to treatment, although very limited information is available. Genetic variability is a measure of genetic differences between individuals and between populations of the same species. It is based on the fact that within individuals of the same species, there are natural variations in the genome that produce differences at the genotypic and phenotypic levels. The natural variations in the genome that constitute modifications of the DNA sequence in individuals of a species are called polymorphisms [51,52].

### 4.2. Single Nucleotide Polymorphisms (SNPs) in Chronic Myeloid Leukemia (CML)

#### 4.2.1. Single Nucleotide Polymorphisms (SNPs) and Susceptibility to Chronic Myeloid Leukemia (CML)

In contrast to other malignancies, there is limited information available on the influence of SNPs on susceptibility to develop CML. In this study [53,54], a total of 80 SNPs of genes involved in different pathways involved in the pathogenesis of CML (apoptosis, angiogenesis, cell proliferation) and in drug resistance were studied. A total of 170 patients were diagnosed with CML and 182 controls were included. In multivariate analysis, only *BCL2* rs1801018 was significantly associated with CML susceptibility in both individual and haplotype studies. Patients with the *BCL2* risk allele had a 1.3- to 1.7-fold increased risk of developing CML. In a later work [55] by the same group where a total of 2744 patients (671 cases, 2073 controls) were studied, 2 chromosomal loci, 6q25.1 and 17p11.1, were identified to be associated with susceptibility to CML.

#### 4.2.2. Single Nucleotide Polymorphisms (SNPs) and Pharmacogenomics in Chronic Myeloid Leukemia (CML)

Between 25–30% of CML patients do not achieve optimal response to imatinib treatment. One of the potential causes of resistance includes variations in the distribution mechanisms of imatinib from oral intake to the interior of leukemic cells. In this regard, several studies have been published that analyze the impact of interindividual variability in genes involved in drug metabolism and, therefore, in treatment outcomes and aim to identify potential markers in the pharmacokinetics and pharmacodynamics of imatinib. Such markers could predict for each individual the lack of efficacy of the drug or excess drug toxicity, developing personalized treatment guidelines. Most pharmacogenomics studies have been performed on the human organic cation transporter 1 transporter OCT-1, which internalizes imatinib into the target cell BCR::ABL1 [54], and on the adenosine triphosphate binding cassette (ABC) transporter *ABCB1*, which expels imatinib from the interior of hematopoietic cells [55]. The impact of genetic variants of OCT-1 on the absorption and transport of imatinib and, consequently, on the efficacy of the drug has been extensively investigated. However, the different studies published until now show contradictory results [56]. Most of them did not find a statistically significant correlation [57,58,59,60,61] and therefore no OCT-1 genetic variant has been identified as influencing the efficacy of imatinib, which is why its clinical applicability is not possible due to the limited evidence [56]. The conception that OCT-1 is the principal uptake transporter for imatinib comes from in vitro studies which showed that some agents known to inhibit OCT-1 also inhibited imatinib uptake into leukemic cells. These experiments did not prove that imatinib was transported by OCT-1. Other studies used various OCT-1-expressing cell models well-established to study OCT-1-mediated transport. One found that OCT-1 expression in oocytes did not promote imatinib uptake. This observation was confirmed by studies performed with OCT-1-transfected mammalian cells which showed that substantial overexpression of functional OCT-1 protein does not result in imatinib transport. Another key finding of the study was that neither the CML cell lines nor the CD34+ CML cells express OCT-1 protein. The conclusion was that OCT-1 does not transport imatinib and imatinib accumulation into leukemic cells occurs independently from OCT-1. Due to the different results of the various studies, mechanisms responsible for imatinib uptake into leukemic cells are still elusive and need further study [62].

Concerning *ABCB1* (MDR1), imatinib is a substrate of P-protein-mediated efflux, which in turn is encoded by this gene. Variation in the function of this gene could explain the variability of responses to treatment, as SNPs in ABCB1 could alter the function of the protein and thus influence drug absorption and elimination [63]. Some studies have demonstrated increased *ABCB1* expression in the advanced stages of CML, as well as the relationship between higher *ABCB1* expression and a lower rate of imatinib resistance [64,65,66]. The three most frequent SNSs are 1236T/C, 2677G>T/A, and 3435C/T. Other studies have demonstrated significantly higher rates of major molecular response (MMR) to imatinib in patients with 1236TT or 2677TT/TA, while some others have reported reduced rates of MMR and complete molecular response [64,67]. Nevertheless, the studies have not been able to find a strong correlation to affirm the association between the levels of *ABCB1* expression with imatinib responsiveness [64].

The *ABCG2* is a constitutively expressed ATP-binding cassette transporter that protects against xenobiotic molecules with a unique structure and function. Also known as breast cancer resistance protein (BCRP), *ABCG2* is a multi-drug transporter with physiological roles in many tissues, including the mammary gland and the blood-brain, blood-testis, and maternal-fetal barriers. *ABCG2* transports uric acid and plays an essential role in the pathology of gout. Similar to its functional homologues *ABCB1* and *ABCC1*, *ABCG2* also has a notorious function in extruding antitumor drugs from various cancer cells, which can result in multidrug resistance, a severe obstacle in cancer treatment [68,69]. A recent study reported structures of *ABCG2* in complex with the anticancer drugs imatinib and mitoxantrone. However, it remains unclear which residues are involved in specific drug-*ABCG2* interactions and how larger drugs can be accommodated and transported by *ABCG2* [70]. The *ABCG2* expression appears to be involved in TKI resistance. The *ABCG2* 34G/A and 421C/A variants were studied in Malaysian CML patients and found to have a better response to imatinib in carriers of the A34A421 dipotype [71]. A meta-analysis of 14 studies showed a markedly superior MMR and complete cytogenetic remissions in CML patients carrying the 421A variant [72].

Bruzzoni-Giovanelli et al. [73] identified an *ABCG2* haplotype (defined as G-G, rs12505410, and rs2725252) associated with a significantly higher cumulative incidence of major molecular response (CI-MMR) in the 400 mg/d imatinib treated patients. Patients carrying this *ABCG2* haplotype in the 400 mg arm were observed to achieve similar CI-MMR rates as patients randomized to the 600 mg/d imatinib arm. Thus, response to imatinib may be influenced by constitutive haplotypes in drug transporter genes.

At present, it is not possible to anticipate which patients in deep molecular response will relapse if TKI treatment is discontinued. Remarkably, a recent study found an association between *BCL2L11* gene polymorphisms (BIM) and the risk of relapse after discontinuation of imatinib [74]. Thus, patients in deep molecular response carrying the *BCL2L11* deletion polymorphism had a higher risk of relapse than in other cases. Confirming these results in other series could be of great clinical interest [75]. A study identified a cohort of imatinib-treated patients with the highest risk of treatment failure, disease progression, and low likelihood of MR by combining the SOKAL risk score and heritable genetic biomarkers, including BIM. However, the utility of this risk score should be determined in prospective clinical trials [76,77].

*ERCC1* gene polymorphisms were significantly associated with response to imatinib treatment. Thus, there was a higher rate of CCR and MMR in those patients with TT genotype compared to those with CT and CC [78].

It is noteworthy that the increased risk of hyperbilirubinemia is caused by nilotinib, because it inhibits human uridine diphosphate-glucuronosyltransferase (*UGT1A1*) activity. Several studies describe the relationship between the *UGTA1* genetic polymorphism and the increased risk of hyperbilirubinemia. The study by Abumiya et al. [79], includes 34 Japanese patients with imatinib-resistant or newly diagnosed CML and demonstrates that the ratio of nilotinib concentration(C0) to total bilirubin levels in CML patients with *UGT1A1* *6/6* or *6/*28 genotypes was higher than those with *UGT1A1* *1/*1, *1/*6, *1/*28, or *27/28* genotypes. As a result of hyperbilirubinemias, the daily dose of nilotinib for patients with UGT1A1*6/*6 or *6/*28 genotypes is approximately 300–400 mg/day, lower than for patients with *UGT1A1* *1/*1, *1/*6, *1/*28, or *27/*28 genotypes (600 mg/day). In this study, all patients who received a lower dose of nilotinib achieved a higher molecular response (MMR) at 12 months, which is predictive of longer progression-free survival. Another study by Shibata et al. [80] also shows that *UGT1A1* polymorphisms are important determinants of severe nilotinib toxicity in Japanese patients.

## 5. General Acute Myeloid Leukemia (AML) Pharmacogenetics

Advances in the biological understanding of AML have identified a significant number of potential therapeutic targets. The requirement for morphological, immunophenotypic, cytogenetic, and molecular analysis highlights these therapeutic targets. All of these have led to intense pharmacological research and development, which has now led to the availability of new drugs.

Immunophenotyping by flow cytometry allows us to monitor MRD. It is a good tool for diagnosing and classifying the disease, as we can detect different antigens that identify the different hematopoietic cell lines and stages of maturation. There is a wide panel of monoclonal antibodies specific for myeloid antigens, including CD13, CD33, CD11b, CD15, CD14, CD36, GA, HLA-DR, TdT, CD34, and CD9.

Cytogenetic and molecular techniques are essential for diagnosis, subtype categorization, individualization of treatment, and prognosis (Table 4). Thus, we can distinguish AMLs with normal karyotypes, which comprise 40–50% of all AMLs, and those with unfavorable prognosis or high risk, which correspond to 10–30%. At the molecular level, class I mutations affect genes that regulate cell proliferation, such as FLT3 mutations and other genes such as JAK2, NRAS, or cKIT. This group also includes mutations in the CEBPα gene, which are considered to have a good prognosis, and others, such as molecular mutations in the *RUNX1* gene, which have an adverse prognosis. Class II mutations are those affecting transcriptional regulatory genes, such as the CBF group mutations, which primarily manifest as translocations detectable by fluorescence in situ hybridization (FISH), such as t(8;21), inv(16), and t(15;17), the latter being responsible for the *PML-RAR-α* gene fusion characteristic of acute promyelocytic leukemia (APL). Class III mutations include those of the MLL gene and different chromatin-modifying genes, all of which have a poor prognosis, and mutations in DNA methylation genes, such as IDH1/2, which have a favorable prognosis. Finally, class IV mutations such as those in the nucleophosmin gene *NPM1* confer a favorable or intermediate prognosis. Many of these molecular markers are currently being studied, and some have become a therapeutic target [81].

## 6. Clinical Application of TKIs in CML

At present, the study of genetic polymorphisms in genes involved in metabolism, transport, proliferation, and apoptosis is one of the most interesting research lines to identify biomarkers associated with CML.

### 6.1. Mutations in BCR::ABL1

The kinase domain of the BCR::ABL1 protein extends from amino acid (Aa) 242 to 493. The site of TKI binding is key to the protein’s function. Mutations in the ABL kinase domain of *BCR::ABL1* are the most common and best-known cause of TKI resistance. Their origin is unclear, although there are indications that they are a consequence of gene instability caused by the presence of *BCR::ABL1* [82,83]. More than 100 mutations with more than 90 Aa changes have been described, and new mutations continue to be described with the use of new inhibitors. Their prevalence depends on the stage of the disease and the sensitivity of the method used to study them. They are much more frequent in late-stage disease and especially in secondary resistance [84]. The most frequent mutations are T315I and E255V, which account for 30–40% of all mutations. The following list includes 90% of all mutations: M244V, L248V, G250E, Y253F/H, E255K/V, T315I, F317L, M351T, E355G, F359C/V, and H396R/P. The polyclonal mechanism of appearance means that it is possible to find one clone with several mutations, several clones with different mutations, or the sequential appearance of several mutations over time [85].

With imatinib mesylate, the P-loop mutations have a poor prognosis, and T315I confers absolute resistance. In general, the appearance of a mutation after treatment with imatinib mesylate confers a slightly lower response rate to the second inhibitor.

With second and third-generation inhibitors, the presence of the T315I mutation also confers complete resistance to dasatinib, nilotinib, and bosutinib, so these are not recommended for use as this clone is being selected. In contrast, ponatinib is active in CML with this mutation [85].

With the use of new TKIs, new mutations appear due to the positive selection they exert on clones. This is the case of the F317L or V299L mutations with dasatinib. Some mutations are more sensitive to some inhibitors than others. This is an example of the P-loop mutations slightly more sensitive to dasatinib. Y253H, E255K/V and F359C/V are insensitive to nilotinib and E255K and V299L to bosutinib. If a patient has F317L/V/I/C, V299L, or T315A mutations, nilotinib is used rather than dasatinib. If, on the other hand, they have Y253H, E255K/V, or F359V/C/I mutations, dasatinib is recommended over nilotinib [84]. Ponatinib is a new third-generation TKI that is highly effective in patients carrying the T315I mutation, achieving up to 60% cytogenetic responses [85].

Pharmacokinetically, TKIs are characterized by oral administration, moderate bioavailability, a large volume of distribution, high plasma protein binding, and elimination after metabolism by CYP 450, which is reflected in Table 5.

### 6.2. Plasma and Intracellular Levels

#### 6.2.1. Plasma Levels

Imatinib Mesylate: dose-dependent, but it shows remarkable interindividual variation. A plasma value (Cmin) above 1000 ng/mL (approximately the mean and median value for IM 400 QD) is associated with a higher rate of complete cytogenetic response (CCR) and major molecular response (MMR) [91,92,93]. It has been suggested that the adverse prognosis of low plasma levels only applies to patients with low OCT-1 activity [94].

The relationship of high plasma level to toxicities is not consistent; only some toxicities, such as oedema, rash, arthralgia, and myalgia, are associated with imatinib Cmin [93,94,95], hyperbilirubinemia and increased lipase (but not alanine aminotransferase or aspartate aminotransferase) with NI Cmin, increased QTcF with nilotinib [96] Cmin and Cmax, pleural effusion with dasatinib [97] Cmin, or diarrhea with bosutinib [98] AUC and Cmax.

#### 6.2.2. Intracellular Level and Pharmacogenetics

The main TKI transporters are the organic cation transporter member 1 (OCT-1, *SLC22A1* gene), the P-glycoprotein (P-gp, *ABCB1* gene), and the breast cancer resistance protein (*BCRP*, *ABCG2* gene) [84]. These transporters are involved in the entry and excretion of TKIs in intestinal cells, hepatocytes, the bile duct, the renal tubule, and brain tissues, thus determining the absorption and excretion of TKIs and ultimately the plasma and tumor cell concentration of the TKI [84].

OCT-1: The entry of imatinib mesylate into the leukemic cell is an active phenomenon dependent on OCT-1, which may be involved in resistance to IM. Patients treated with MI at 400 mg and with low OCT-1 activity have a lower probability of achieving MMR and even lower event-free survival, progression-free survival, and 5-year overall survival. It is unclear whether IM at 600–800 mg completely overcomes the adverse prognostic effect of low OCT-1 activity [94]. Some studies have associated specific OCT-1 single nucleotide polymorphisms (SNPs), such as M420del, with suboptimal response to MI IM and low OCT-1 activity [92,93,94,99]. Although Nies et al. concluded that OCT-1 did not transport imatinib into primitive leukemic cells and corroborated previous studies demonstrating that OCT-1 activity varied in primary CML cells, the study did not investigate its relevance as a biomarker. There are different opinions due to inconsistencies in the conclusions from studies investigating common OCT-1 variants. Polymorphism in genes influencing imatinib absorption and transport, such as OCT-1, are postulated to impact imatinib’s bioavailability and the observed patient response. Despite this, and given the significant disparity of the results, the role of OTC-1 in imatinib action is still uncertain [57]. This finding could facilitate OCT-1-based clinical trials and their clinical application. NI, DA, BO, PO, and AS are relatively permeable and do not depend on OCT-1 for entry into the leukemic cell.

## 7. Clinical Application in AML

Although anthracycline and cytarabine combination regimens remain effective in most patients, the variability of outcomes has been primarily associated with the genetic variability of these patients. Several pharmacogenetic studies have analyzed toxicities or health outcomes of polymorphisms in genes encoding transporters, metabolizers, or molecular targets of chemotherapeutic agents [100]. The different pharmacokinetic and pharmacodynamic biomarkers are grouped according to the pharmacological group to which they belong. Thus, the drugs used in AML on which we will focus are anthracyclines, cytarabine, midostaurin, and gemtuzumab ozogamicin (Table 6).

*Anthracyclines:* drugs characterized by cardiotoxicity. They can cause congestive heart failure and cardiomyopathy, with dose accumulation being one of the most important risk factors. However, episodes of cardiotoxicity have been observed in patients with unknown risk factors caused by an idiosyncratic reaction. Factors influencing the disposition of anthracyclines in cells and tissues include transporters encoded by ABC family genes. ABCC3 variants in other pathologies have been associated with lower SLE, and ABCG2 variants rs2231137 and rs2231142 with an increased risk of cardiac and pulmonary toxicities [101,102]. The main enzymes responsible for anthracycline metabolism are CBR and AKR. Several studies have associated the CBR1 variants rs1143663, rs20572, rs9024, and rs41557318 with decreased metabolism and increased cardiotoxic potential, while the CBR3 variant rs1056892 has been associated with increased anthracycline metabolism [102,103]. All these variants are listed in one of the main pharmacogenetic databases, PharmGKB^®^, and all of them have a level of evidence equal to or less than 3.

*Cytarabine:* Cytarabine is a nucleoside analogue that interferes with DNA synthesis and has the main toxic effects of nausea, vomiting, diarrhea, abdominal pain, and bone marrow suppression. Cytarabine uptake into cells is mediated by the *SLC29A1* gene, encoding hENT1, which is responsible for transporting about 80% of cytarabine. Different levels of *SLC29A1* expression have been observed, and patients with hENT1 deficiency had lower EFS and OS [103]. To be active, cytarabine needs to be metabolized by deoxycytidine kinase (DCK) and other kinases, with DCK being the enzyme that catalyzes the first phosphorylation step and limits the rate of activation. Several DCK variations have been reported, most notably rs80143932 and rs2306744, which appear to be associated with increased SLE in Asian populations [104]. However, no correlation has been observed between DCK expression and enzyme activity. Polymorphisms of other genes such as *CMPK1*, *NME1*, *NT5C2*, *NT5C3*, *CDA*, *DCTD*, *CTPS*, *RRM1*, or *RRM2* have also been analyzed. Still, most of them have not shown significant clinical relevance [102], and in the Pharmacogenetics database of PharmGKB^®^, they have a grade of evidence equal to or lower than 3.

*Midostaurin:* Among the mutations designated as type I mutations, a mutation in the *FLT3* gene is present in approximately 30% of newly diagnosed AML. FLT3 is a receptor tyrosine kinase with a significant role in the differentiation, proliferation, and apoptosis of haematopoietic cells. Its activation can result either from internal tandem duplication of amino acids within the juxtamembrane domain of the receptor (FLT3-ITD) or from point mutations in the second tyrosine kinase domain (FLT3-TKD). Approximately 65% of mutated patients have the ITD subtype, which is associated with a worse prognosis (high relapse rate). About 8% of patients have the TKD subtype, which has a controversial but more favorable prognosis [102]. In previous studies, the rs1933437 variant has been reported to be associated with toxicity in treatment with sunitinib [105]. Midostaurin inhibits FLT3 receptor signal transduction and induces cell cycle arrest and apoptosis in leukemic cells expressing both ITD and TKD subtypes. Combination therapy has been shown to be superior to standard conventional therapy in the pivotal RATIFY clinical trial, which showed an increase in overall survival (OS) with a decrease in the risk of death of 23% (HR: 0.77) and an increase in event-free survival (EFS), with an increase in the proportion of long-term survivors of 7–8% [106]. No polymorphisms implicated in potential toxicities or health outcomes have yet been reported for midostaurin.

*Gemtuzumab ozogamicin:* Another therapeutic target that has been studied is the CD33 antigen. It is a single-chain transmembrane glycoprotein that is expressed on the surface of normal and leukemic myeloid cells, being present in more than 80% of leukemic cells but absent from pluripotent stem cells. Gemtuzumab ozogamicin is a monoclonal antibody directed against CD33 covalently linked to a semi-synthetic cytotoxic agent. It is used in the treatment of patients with de novo CD-33 positive AML with favorable or intermediate cytogenetic risk in combination with standard chemotherapy. Regarding pharmacogenetic variants, the CD33 rs35112940 variant is implicated in clinical outcomes in pediatric patients [107] and the organic cation transporter SLC22A12. The wild-type homozygote rs11231825 is associated with increased infusional drug reactions. On the other hand, the variant allele of *SULT2B1* rs2302948 was associated with a protective factor [108]. In the pivotal ALFA-0701 clinical trial, gemtuzumab ozogamicin demonstrated an increase in EFS versus chemotherapy alone (17.3 versus 9.5 months, HR: 0.652) in previously untreated AML patients aged 50–70 years [109].

Finally, new drugs are being studied, including farnesyltransferase inhibitors, second-generation FLT3 inhibitors such as gilteritinib, quizartinib, and crenolanib, bcl-2 antisense oligonucleotides such as venetoclax, new hypomethylating drugs, daunorubicin and cytarabine as liposomal formulations, and histone deacetylases inhibitors (HDI) such as enasidenib and ivosidenib.

**Table 6:** Summary of Major Genetic Polymorphisms and their clinical impact in AML.

Gene	Snp (rs)	Drug	Level of Evidence	Clinical Application	Clinical Impact (Toxicity/Effectiveness)	Reference
*CBR1*	rs20572	Doxorubicin	3	No	Toxicity	[110]
*CBR1*	rs9024	Anthracyclines	3	No	Toxicity	[110]
*CBR1*	rs1056892	Anthracyclines	3	No	Toxicity	[110]
*DCK*	rs80143932	cytarabine	3	No	Effectiveness	[104]
*DCK*	rs2306744	cytarabine	3	No	Effectiveness	[104]
*CD33*	rs35112940	Gemtuzumab ozogamicin	3	No	Effectiveness	[107]
*SLC22A12*	rs11231825	Gemtuzumab ozogamicin	3	No	Toxicity	[104]
*SULT2B1*	rs2302948	Gemtuzumab ozogamicin	3	No	Toxicity	[104]

## 8. Conclusions

The precision medicine achieved through implementing Pharmacogenetics should be a reality in daily clinical practice in Oncohematology. Its implementation represents a definite improvement in treating acute and chronic myeloid leukemia patients, favoring the system’s sustainability by selecting patients and avoiding costly and unnecessary treatments. It also minimizes complications resulting from treatments with little or no chance of response to treatment. The ultimate challenge in pharmacogenetic advances in these pathologies would be to have health outcomes and improve the clinical results of oncohematological patients and their quality and quantity of life.

## Figures and Tables

**Table 1:** Comparison of different tyrosine kinase inhibitors (TKIs).

TKI	Primary Targets	Secondary Targets	Therapeutic Indications	Reference
Imatinib Glivec^®^	BCR::ABL1 c-ABL	c-KIT, PDGFR (alpha and beta), DDR 1, DDR 2, NQO2, Arg, CSF-1R	CML Ph (+) (BCR::ABL1) newly diagnosed when bone marrow transplantation is not considered as 1st line treatment. CML Ph (+) in chronic phase after interferon-α treatment failure, accelerated phase, or blast crisis.	[16]
Dasatinib Sprycel^®^	BCR::ABL1 c-ABL	SRC, c-KIT, PDGFR-b, EPHA2, FMS, DDR 1, DDR2	CML Ph (+) (BCR::ABL1) in chronic phase of recent diagnosis. CML Ph (+) (BCR::ABL1) in chronic, accelerated, or blast phase with resistance or intolerance to previous treatment, including imatinib.	[17]
Nilotinib Tasigna^®^	BCR::ABL1 c-ABL	c-KIT, PDGFR (alpha and beta), DDR 1, NQO2, VEGF, r ephrin, ZAK	CML Ph (+) (BCR::ABL1) of recent diagnosis in chronic phase. CML Ph (+) (BCR::ABL1) in chronic, accelerated, or blastic phase with resistance or intolerance to previous treatment, including imatinib. No effectiveness data are available in patients with blast phase CML.	[18]
Bosutinib Bosulif^®^	BCR::ABL1 c-ABL	SRC, c-Fms, EphA, t ephrin B, Trk, Axl, Tec, Ste20, serine/threonine, others	CML Ph (+) (BCR::ABL1) of recent diagnosis in chronic phase. CML Ph (+) (BCR::ABL1) in accelerated or blastic phase previously treated with one or more tyrosine kinase inhibitors and for whom imatinib, nilotinib, and dasatinib are not considered to be suitable options.	[19]
Ponatinib Iclusig^®^	BCR::ABL1 c-ABL	SRC, c-KIT, PDGFR, VEGFR, FGFR, RET, FLT3	CML in a chronic, accelerated, or blastic phase that is resistant or intolerant to dasatinib or nilotinib; in whom subsequent treatment with imatinib is not clinically indicated and those with the T315I mutation.	[20]
Asciminib Scemblix^®^	BCR::ABL1 c-ABL	-	CML Ph (+) (BCR::ABL1) in chronic phase, previously treated with two or more TKIs. It is also indicated in the treatment of CML Ph (+) in adult patients with the T315I mutation.	[15]

**Table 2:** Acute Myeloid Leukemia standard regimen [21,22].

Phases	Objective	Treatment Scheme
Induction phase	To achieve CR	1 or 2 cycles of anthracyclines (3 days) + cytarabine (ARA-C) (7 days).
Post-remission phase	To eradicate the residual leukemic clone	Consolidation stage: One cycle equal to induction phase +2 or 3 cycles of intensification (intermediate or high dose of cytarabine). When first CR is achieved: Autologous or allogeneic hematopoietic stem cell transplantations (SCT) of patients with high risk or relapse. Exception: Patients who are not candidates for intensive therapies should be approached on an individual bases.
Treatment alternatives		Low dose of ARA-C. Use of hypomethylating agents: 5-azacitidine or decitabine. Inclusion of the patient in clinical trials: new cytotoxic agents or supportive therapy with chemotherapy and general care.

**Table 3:** New alternative treatment in Acute Myeloid Leukemia.

Treatment			Targets		Therapeutic Indications		Limitations
FMS-like tyrosine kinase 3 (FLT3) inhibitors	First generation	Type II: sorafenib	FMS-like tyrosine kinase 3 (FLT3)	Other kinases (KIT, PDGFR, RAS/RAF/MEK, JAK)	Effective only in ITD	25–35% of de novo AMLs have mutations in FLT3, both internal tandem duplications (ITD) and point duplications (TKD). The preferred approach is a combination of FLT3i with intensive chemotherapy in fit/young patients or with an HMA ± venetoclax in older or unfit patients with overall responses reaching 40–50%. They also show great promise as post-transplant maintenance to reduce the risk of relapse [26,27].	Many studies have shown that FLT3 inhibitors have favorable clinical activities for AML patients with FLT3/ITD, but response duration remains short because of the rapid development of resistance. Resistance to FLT3 inhibitors was attributed to the emergence of new mutations. The secondary FLT3 tyrosine kinase domain (TKD) mutation was one of the new mutations in the patients who showed resistance to FLT3 inhibitors [28,29].
		Type I: lestaurtinib, sunitinib, midostaurin			Effective in ITD and TKD		
	Second generation	Type I: crenolanib, gilteritinib		-More potent and specific. -Less off-target effects.	Effective only in ITD		
		Type II: quizartinib					
CAR-T cells [30]			CD33 (CART 33) and (CART 123).		In many patients with AML, the disease will relapse with a poor prognosis and few options for targeted therapy. Chimeric antigen receptor (CAR) T cells targeting myeloid-lineage antigens such as CD33, and CD123 β have produced promising results in preclinical models of AML.		Circulating CAR-T cells are unable to differentiate between normal and malignant cells. This often induces on-target off-tumor side effects such as B-cell aplasia that can be treated by monthly intravenous immunoglobulin infusions. This represents a major obstacle to the development of CAR T-cell therapy in AML.
Venetoclax [31,32,33,34]			B-cell lymphoma 2 (BCL-2): Overexpression of BCL-2 has been shown to exist in AML, where it mediates tumor cell survival and has been associated with antineoplastic drug resistance. Venetoclax binds directly to the BH3 domain binding site of BCL-2, displacing proapoptotic proteins with BH3 domains, such as BIM, to initiate mitochondrial outer membrane permeabilization (MOMP), caspase activation, and programmed cell death.		In combination with a hypomethylating agent (5-azacitidine or decitabine). Some studies showed that the addition of venetoclax to the HMA and FLT3 inhibitor combination (triplet therapy) was associated with improved outcome parameters and may be an effective frontline regimen not only in older/unfit FLT3 mutated AML but potentially also in younger patients with adverse biologic features, although further data from ongoing and planned randomized trials in such populations are needed to support such a paradigm shift.		Despite its promising efficacy, clonal evolution and drug resistance limit the prolonged durability of the responses in AML patients.
Isocitrate dehydrogenase (IDH) inhibitors: Ivosidenib and enasidenib [35,36,37,38,39,40]			IDH: IDH1 and IDH2 mutations result in the aberrant production of 2-hydroxyglutarate, which leads to DNA and histone hypermethylation and impaired myeloid differentiation, promoting oncogenesis in AML. IDH1/2 mutations are found in around 20% of patients with AML.		ivosidenib: For IDH1-mutated AML patients ≥75 y or unsuitable for intensive chemotherapy due to comorbidities. enasidenib: For IDH2-mutated AML patients ≥75 y or unsuitable for intensive chemotherapy due to comorbidities.		Both IDH inhibitors have been associated with a rare but fatal complication termed IDH inhibitor differentiation syndrome (IDH-DS). Signs and symptoms of IDH-DS include dyspnea, unexplained fever for two days, pulmonary infiltrates, hypoxemia, CTCAE ≥ grade 2 acute kidney injury, pleural effusion, arthralgias, lymphadenopathy, skin rash, disseminated intravascular coagulopathy, edema or weight gain of >5 kg, and pericardial effusion. Management consists of the timely initiation of dexamethasone 10 mg IV twice daily and tapered once symptoms improve. IDH inhibitors should be held for severe pulmonary symptoms and renal dysfunction persisting for over 48 h after initiation of dexamethasone or equivalent corticosteroids. Because enasidenib and ivosidenib have long half-lives of elimination, corticosteroids should be initiated as soon as feasible as treatment interruption alone might not result in immediate resolution of symptoms.
Gemtuzumab ozogamicin (GO) [41]			CD33: Anti-CD33 monoclonal antibody linked to a cytotoxic derivative of calicheamicin. The transmembrane surface receptor, CD33, is an attractive target for AML as it is nearly ubiquitously expressed on hematopoietic cells of myeloid lineage and myeloblasts in >80% of AML patients.		Is the only new agent approved for the treatment of the vast majority of AML patients, including ND-AML and RR-AML disease in adults and RR-AML in pediatric patients. Of note, these indications for GO expand upon the previous US approval of GO, which was limited only to adult patients with RR-AML in 2000. A recent study, using fractionated doses of GO combined with ARA-C, demonstrated a CR rate of 75%, and a 2-year OS of 51% with low mortality (8.3%).		Despite the known clinical efficacy in relapsed/refractory acute myeloid leukemia (AML), GO was withdrawn from the market in 2010 due to increased early deaths seen in newly diagnosed AML patients receiving GO + intensive chemotherapy. In 2017, new data on the clinical efficacy and safety of GO administered on a fractionated dosing schedule led to re-approval for newly diagnosed and relapsed/refractory AML.
Glasdegib [42]			Oral small-molecule inhibitor of the Smoothened protein and the Hedgehog signaling pathway.		In combination with low-dose cytarabine (LDAC) was FDA approved in 2018, for patients with AML or high-risk myelodysplastic syndrome (MDS) unsuitable for intensive chemotherapy.		Despite the positive readout, the efficacy of glasdegib plus LDAC appears modest when compared with the data of hypomethylating agent-venetoclax combinations in similar, unsuitable for intensive chemotherapy patient populations, albeit with the caveat and hazards of comparisons across distinct clinical trials. For this reason, azacitidine plus venetoclax appears to have become the favored approach for the frontline treatment of AML in patients unsuitable for intensive therapy.
Volasertib [43,44,45,46,47,48]			Inhibitor of polo-like kinases (PLKs): potently inhibits PLK1 as well as the two closely related kinases, PLK2 and PLK3, with 50% inhibitory concentration values of 0.87, 5, and 56 nmol/L, respectively [43,44].		In patients older than 60 years, AML is associated with a particularly worse prognosis compared to younger patients, both due to intolerance and treatment resistance to chemotherapy. Volasertib offers a newer approach to the treatment of AML. Volasertib, currently in Phase III clinical trials in combination with cytarabine, is reviewed as a promising agent for this patient population with AML, from the viewpoints of potential compliance and efficacy [45,46].		Volasertib inhibits the proliferation of most leukemia cell lines and primary AML cells in vitro. Although PLK1 is over-expressed in a variety of cancer cells, PLK1 is vital for cell proliferation regardless of normal or malignant cells. The combination with more cancer-specific, molecular targeting agents is suitable for the clinical development of PLK1 inhibitors. Further study is required to identify a subset of AML patients with optimal response to volasertib, and the molecules or pathways that associate with the response to volasertib in AML cells [47].
HDAc inhibitors (HDACi): Panobinostat [44,49,50]			HDA: These enzymes are involved in the removal of acetyl groups from histones, so their inhibition causes transcriptional repression and slows hematopoietic differentiation.		HDACi has emerged as a promising therapeutic strategy for cancer therapy. Furthermore, these inhibitors have shown the ability to induce differentiation, cell cycle arrest, and apoptosis in AML, leading to a good alternative for treatment, especially for those AML patients not suitable for intensive chemotherapy.		Despite the promising preclinical results of HDACi, these HDACi do not seem to be clinically effective as monotherapies in AML. However, combination strategies with a variety of anticancer drugs are being tested in clinical trials, showing significant anti-leukemic activity in hematological diseases as they enhance the action of some standard-of-care anti-AML treatments.

FLT3: FMS-like tyrosine kinase 3, ITD: internal tandem duplications, TKD: FLT3 tyrosine kinase domain (TKD), HMA: hypomethylating agents.

**Table 4:** Genetic risk of AML according to the 2017 European Leukaemia Net recommendations. Adapted from [21].

**AML with genetic abnormalities with a favorable prognosis**
t(8;21)(q22;q22.1);*RUNX1*-*RUNX1T1* inv(16)(p13.1q22) o t(16;16) (p13.1;q22);*CBFB*-*MYH11* Acute promyelocytic leukemia t(15;17) (q22;q11-12);*PML-RARA* Mutated NPM1 without *FLT3-ITD* (or FLT3-ITD with a low allelic ratio) Biallelic mutated *CEBPA*
**AML with genetic abnormalities of intermediate prognosis**
Mutated NPM1 and *FLT3-ITD* high Wild-type NPM1 without *FLT3-ITD* or with *FLT3-ITD* low t(9;11)(p21.3;q23.3);*MLLT3-KMT2A*
**AML with genetic abnormalities of unfavorable prognosis**
t(6;9)(p23;q34.1);*DEK-NUP214* t(v;11q23.3); *KMT2A* rearranged inv(3)(q21.3q26.2) or t(3;3)(q21.3;q26.2);*GATA2,MECOM* Wild-type *NPM1* and *FLT3-ITD* high Mutated *RUNX1* Mutated *ASXL1* Mutated *TP53*

**Table 5:** ADME pharmacokinetics properties.

Pharmacokinetics Properties	Imatinib	Nilotinib	Dasatinib	Bosutinib	Ponatinib	Asciminib
Bioavailability	98%	31%	34%	50%	unknown	≈40%
Cmax (median)	2.5 h	3 h	1 h	5 h	4 h	2.5 h
Food	No interaction	High interaction	Minimum interaction	High interaction	No interaction	High interaction
Plasma protein binding	95%	98%	96%	96%	>99%	93%
Metabolism	Hepatic CYP	Hepatic CYP	Hepatic CYP	Hepatic CYP	Hepatic CYP	Hepatic CYP
Half-life	18 h (40)	17 h	5–6 h	32–39 h	22 h	8 h
Excretion	Fecal Urinary 13%	Fecal Urinary 4%	Fecal Urinary 4%	Fecal Urinary 3%	Fecal Urinary 5%	Fecal Urinary 11%
Reference	[86]	[87]	[88]	[89]	[16]	[90]

## Data Availability

Not applicable.
